# Single-cell atlas of endothelial cells in atherosclerosis: identifying C1 CXCL12+ ECs as key proliferative drivers for immunological precision therapeutics in atherosclerosis

**DOI:** 10.3389/fimmu.2025.1569988

**Published:** 2025-05-12

**Authors:** Zhenzhen Zhao, Yujiang Dong, Zhijie Zhao, Zhikai Xiahou, Cong Sun

**Affiliations:** ^1^ College of First Clinical Medicine, Shandong University of Traditional Chinese Medicine, Jinan, China; ^2^ Department of Cardiovascular Disease, The Second Affiliated Hospital of Shandong University of Traditional Chinese Medicine, Jinan, China; ^3^ Department of Plastic and Reconstructive Surgery, Shanghai Ninth People’s Hospital, Shanghai JiaoTong University School of Medicine, Shanghai, China; ^4^ China Institute of Sport and Health Science, Beijing Sport University, Beijing, China

**Keywords:** atherosclerosis, single-cell RNA sequencing, CXCL12, vascular remodeling, inflammation, cellular heterogeneity

## Abstract

**Background:**

Atherosclerosis (AS) is a chronic inflammatory disease characterized by endothelial dysfunction, monocyte infiltration, smooth muscle proliferation, and extracellular matrix accumulation. Endothelial cell (EC) dysfunction plays a pivotal role in the initiation and progression of AS. Despite progress in traditional research methods, the complexity of cellular heterogeneity within the disease remains poorly understood, necessitating a more refined approach for uncovering disease mechanisms.

**Methods:**

In this study, we employed single-cell RNA sequencing (scRNA-seq) to map the endothelial cell landscape in AS comprehensively. By analyzing cellular heterogeneity, differentiation trajectories, and functional states, we identified critical endothelial subpopulations and their roles in the progression of AS. Functional enrichment and differentiation analyses were conducted, and the findings were validated through *in vitro* experiments.

**Results:**

The single-cell analysis revealed distinct EC subpopulations with unique contributions to AS progression. Among these, C1 *CXCL12*+ ECs emerged as a key subpopulation associated with endothelial differentiation, vascular remodeling, and inflammation. These cells demonstrated high proliferative potential and were enriched in pathways related to endothelial migration and repair. Through CCK-8, Transwell assay, EdU staining and angiogenesis ability, we found that knockdown of FOXM1 in C1 CXCL12+ ECs resulted in decreased proliferation, migration and invasion. Thus, it affects the progression of AS.

**Conclusion:**

This study provides a detailed single-cell atlas of endothelial cells in AS, identifying critical subpopulations, regulatory pathways, and key factors driving disease progression. The application of single-cell technologies paves the way for advancing our understanding of cardiovascular diseases and offers significant potential for developing personalized therapeutic strategies in immunology and precision medicine.

## Introduction

Atherosclerosis (AS) is a chronic inflammatory disease with high heterogeneity. Its main features include monocyte infiltration, smooth muscle proliferation, matrix protein accumulation in intima and endothelial dysfunction (1). The dynamic changes of endothelial cells (ECs) play a key role in the occurrence and development of AS (2). After endothelial injury, the arterial wall becomes a lipid load and undergoes a process similar to wound healing, resulting in characteristic morphological changes (3, 4).

One of the early changes in the development of AS is the activation and dysfunction of ECs in the vulnerable area of arterial blood vessels (5). At this time, ECs showed the phenotype of promoting inflammation and thrombosis, and its barrier function was damaged, which was mainly caused by hemodynamic disorder. Endothelial dysfunction in areas prone to vascular diseases is an important factor in the pathobiology of atherosclerotic cardiovascular diseases (6). ECs dysfunction, broadly speaking, refers to the non-adaptive changes of EC phenotype, which affects hemostasis, thrombosis, local vascular tension, redox balance and the regulation of acute and chronic inflammatory reactions in arterial wall (7). Endothelial activation (expression of adhesion molecules and chemokines) in the early stage, cell apoptosis and barrier function destruction in the late stage (8, 9). Endothelial injury is usually caused by oxidized lipids, free radicals, cytokines, hemodynamic stress and increased blood cholesterol concentration. These factors can lead to apoptosis or necrosis of EC layer, accompanied by the loss of antithrombotic properties, thus promoting the formation of AS (10).

Single cell technology has made a breakthrough in the field of life science in recent years. This technique can reveal cell heterogeneity, differentiation trajectory and functional characteristics of specific subgroups at the single cell level by high-throughput sequencing of genome, transcriptome, protein group and epigenetic information of a single cell. Compared with traditional population sequencing, single cell technology can overcome the problem that individual differences in cell populations are averaged, which provides a new perspective for understanding the cell composition and dynamic changes of complex tissues. In recent years, single cell technology has played an important role in cardiovascular research (11). Cardiovascular disease is one of the main causes of death and disability worldwide, among which AS, as a chronic inflammatory disease, involves complex cellular interaction and molecular mechanism in the occurrence and development of the disease (12). Through single cell RNA sequencing (scRNA-seq) technology (13, 14), researchers can analyze the cellular heterogeneity, gene expression profile and cell-cell interaction of atherosclerotic lesions, which provides a powerful tool for exploring the pathogenesis and potential therapeutic targets of AS (11).

AS is a complex pathological process involving multiple cells, and its main features include lipid deposition, inflammatory reaction and fibrosis. Although traditional research methods reveal many key molecules and pathways, it is still difficult to fully understand its mechanism (15) due to the lack of accurate analysis of cell heterogeneity. The application of single cell technology enables researchers to analyze the gene expression patterns, functional states of different cell types in the core area (AC) and adjacent areas (PA) of AS and their roles in disease progression (16). For example, single cell sequencing technology can not only identify new cell subpopulations, but also reveal their functions in proliferation, differentiation and immune regulation.

At present, it has been found that ECs, smooth muscle cells (SMCs) and immune cells are important participants in AS (17). Through single cell sequencing technology, researchers have identified a variety of EC subpopulations related to AS, and the functional characteristics of these subpopulations are closely related to the disease progress, such as vascular inflammation, endothelial barrier function and vascular remodeling (18). In addition, single cell technology can also predict the differentiation trajectory and state transition of cells, which provides a direction for exploring new therapeutic strategies.

This study provides a comprehensive single-cell landscape of ECs in AS, analyzes the proliferation ability, differentiation level and functional enrichment of cell subpopulations, and reveals the key subpopulations, pathways and regulatory factors related to disease progression. In addition, the value-added and functional characteristics of key subgroups were verified by *in vitro* experiments. These insights contribute to a deeper understanding of AS biology and may guide the development of targeted therapy for this common vascular disease. Cell sequencing technology has important application potential in the research of cardiovascular diseases and AS. With the further development of technology in the future, it will provide stronger support for revealing disease mechanism, developing new treatment strategies and realizing personalized treatment.

## Materials and methods

### Single-cell data acquisition and processing

AS scRNA-seq (19–21) data were obtained from Gene Expression Omnibus database GSE159677. gene expression analysis was carried out with Seurat in R. Strict quality control filtered out low-quality cells, selecting those with nFeature counts between 300 and 5000, nCount between 500 and 50,000, and limiting mitochondrial and red cell gene expression to less than 10% and 5%, respectively. After filtering, 45,690 cells remained. Since we used data from publicly accessible databases, this study did not require ethical approval.

### Cell subpopulation identification

The NormalizeData functions of Seurat were used to normalize the data. FindVariableFeatures was used to identify the 2000 highly variable genes (HVGs) (22). The scaleData function standardized the data (23–25). Principal component analysis (PCA) (26–28) was performed on these HVGs using the RunPCA function, and Harmony was applied to reduce batch effects. CellCycleScoring calculated the cell cycle phases. For clustering the reduced data, the FindNeighbors and FindClusters functions were used. Uniform Manifold Approximation and Projection (UMAP) (29–31) was used for dimensionality reduction clustering analysis, and the results were displayed in a two-dimensional space. To enhance annotation accuracy, FindAllMarkers was applied alongside reference datasets from the CellMarker database and published literature for single-cell annotation.

### AUcell analysis

The AUCell method of cell stemness assessment identified cells with active gene expression in single-cell RNA-seq data by analyzing gene profiles and using gene sets to determine the level of “activity” in each cell. This study utilized it to assess stem cell properties with different cell subpopulations.

### Enrichment analysis

Gene Ontology (GO) (32, 33) enrichment analysis was a bioinformatics method used to analyze gene function (34–37). Based on the GO database, it mapped a set of genes to three levels: Biological Process (BP), Molecular Function (MF) and Cellular Component (CC) (38). To help identified the functional properties of genes. We used FindAllMarkers in Seurat to detect differentially expressed genes (DEGs) (39, 40) with a Wilcoxon test (min.pct = 0.25, logfc.threshold = 0.25) (41). The DEGs were then analyzed for functional enrichment using ClusterProfiler (v4.6.0) to identify significantly enriched GO terms (adjusted p-value < 0.05) (42). Gene Set Enrichment Analysis (GSEA) (43, 44)was performed using the GSEA tool (http://www.gsea-msigdb.org) (45), which ranked DEGs to find statistically significant and consistent pathway differences between groups (46). Additionally, GSEA was applied to explore biological pathways enriched in each cell cluster based on DEG expression profiles (47).

### pySCENIC analysis

pySCENIC was a Python tool for single-cell transcription factor Gene Regulatory Network (GRN) inference and cell state characterization (48). Firstly, the GRN was calculated to infer the regulatory relationship between transcription factors (TFs) (49) and target genes. Then, the cell state inference method was used to predict the developmental trajectory and state of each cell. Finally, the AUCell algorithm was used to assess the activity of TFs in different cells and quantify their regulatory effects. Through these steps, we could fully understand the role and regulatory mechanism of TFs in single cells.

### Metabolic analysis

Single-cell metabolic analysis was a cutting-edge approach used to study the metabolic processes at the single-cell level (50). Unlike traditional bulk analysis, which averaged gene expression and metabolic activity across many cells, single-cell analysis enabled the identification of metabolic heterogeneity within a population of cells, providing a deeper understanding of cellular functions, disease mechanisms, and cell-to-cell variability in metabolism.

### Infer cell developmental trajectories

CytoTRACE estimated the developmental potential or “stemness” of individual cells (51–53). By analyzing gene expression data, CytoTRACE identified how far along a cell is in its differentiation process, helping to track transitions between different cell states.

Monocle2 was a tool used to order single cells along a trajectory, representing their progression through different stages of differentiation or development. It used dimensionality reduction to map cells into a low-dimensional space and calculates pseudotime, which could reveal gene expression changes as cells progress through developmental pathways, including branching decisions. It used dimensionality reduction to map cells into a low-dimensional space and calculates pseudotime, which could reveal gene expression changes as cells progress through developmental pathways, including branching decisions.

Slingshot focused on identifying cell lineage trees by analyzing clusters of cells (54–56). It built a tree-like structure to represent different cell fates and lineage branches. While it doesn’t calculate pseudotime directly, it was useful for identifying and mapping branching patterns in cell populations that are clustered in a single cell. populations that are clustered in high-dimensional spaces.

### Intercellular crosstalk analysis

The CellChat package enabled the detailed analysis and inference of intercellular communication from scRNA-seq data (57)CellChat applied a mass-action-based model to estimate communication probabilities between two cell groups, considering ligand-receptor interactions, multi-subunit structures (58–60). It also utilized machine learning methods and quantitative indicators for a comprehensive and it also utilized machine learning methods and quantitative indicators for a comprehensive and comparative analysis of cellular communication across different conditions. expressed signaling genes were detected using the Wilcoxon rank sum test.

### Cell culture

Human umbilical vein endothelial cells (HUVECs) were cultured in ECM complete medium (ScienCell, USA) in a constant temperature incubator at 37°C with 5% CO_2_. siRNA transfection was performed when the cell fusion reached 50%-70%. Using Lipofectamine RNAiMAX transfection reagent (Invitrogen, USA), the siRNA and transfection reagent were diluted in serum-free Opti-MEM medium, left at room temperature for 5 minutes, mixed, and incubated at room temperature for 20 minutes to form transfection complexes. It was then added to the cell culture medium.

FOXM1-targeting siRNA consists of two separate siRNA sequences to ensure knockdown specificity: si-1: AAGAAGAAAUCCUGGUUAA, si-2: ACUAUCAACAAUAGCCUAU. The negative control group (si-NC) and FOXM1 knockdown group were set up. Cells were collected 48 hours after transfection for subsequent experiments, including qRT-PCR verification of knockdown efficiency and downstream functional studies. The mRNA levels of FOXM1 were measured by quantitative real-time PCR (qRT-PCR) using the following specific primers: forward primer(F): AAACCTGCAGCTAGGGATGT and reverse primer(R): AGCCCAGTCCATCAGAACTC-.

### RNA extraction and quantitative real-time PCR

QPCR (61, 62) was used to detect gene expression or DNA copy number. RNA was extracted and reverse transcribed into cDNA, and amplified by adding primer and SYBR Green or probe. The relative expression was calculated by Ct value and normalized by internal reference gene. The method was sensitive, specific and suitable for gene quantitative research (63).

### Cell viability assay

To determine cell viability, DOJINDO Cell Counting Kit-8 (CCK-8) was used. Cells were seeded at 1×10^3 cells per well in 96-well plates and cultured overnight. A 100 µL detection reagent was added to each well and incubated for 1 hour. Over the course of 4 days, absorbance at 450 nm was measured daily, and growth curves were plotted by correlating OD450 values with time.

### Edu analysis

In the Edu experiment, cells were incubated with a culture medium containing 10 µM Edu for 30 minutes to 2 hours, fixed and permeabilized, and then the Click reaction mixture was added to mark DNA synthesis. After staining, the cell proliferation activity was observed by fluorescence microscope, and DAPI staining was used to assist the analysis.

### Flow cytometry for apoptosis analysis

Apoptosis was detected by Annexin V-FITC and PI staining in flow cytometry. After the cells were treated, they were washed with PBS and stained. Annexin V labeled early apoptotic cells and PI labeled late apoptotic or necrotic cells. Cell staining was analyzed by flow cytometry to distinguish healthy, early apoptotic and late apoptotic/necrotic cells.

### Transwell migration and invasion assays

Cells were inoculated in a Transwell chamber (64, 65). For the migration experiment, cells were added to the upper layer of the medium, and the medium containing attraction factors was added to the bottom layer (66). For the invasion experiment, Matri-gel was added to the chamber to simulate the matrix. After 24–48 hours, the non-migrating cells were removed, the penetrated cells were fixed and stained, and the number of penetrated cells was counted.

### Angiogenesis assay

In the experiment of endothelial angiogenesis, HUVEC cells were inoculated in Matrigel which was heated to room temperature in advance and cultured for 48 hours. By adding growth factors such as VEGF to induce angiogenesis, the tubular structure of cells was observed. Microscopic imaging was used to analyze the length and number of tubes to evaluate the angiogenesis ability of ECs.

## Results

### Difference analysis of AS EC subpopulations

**Figure 1:**
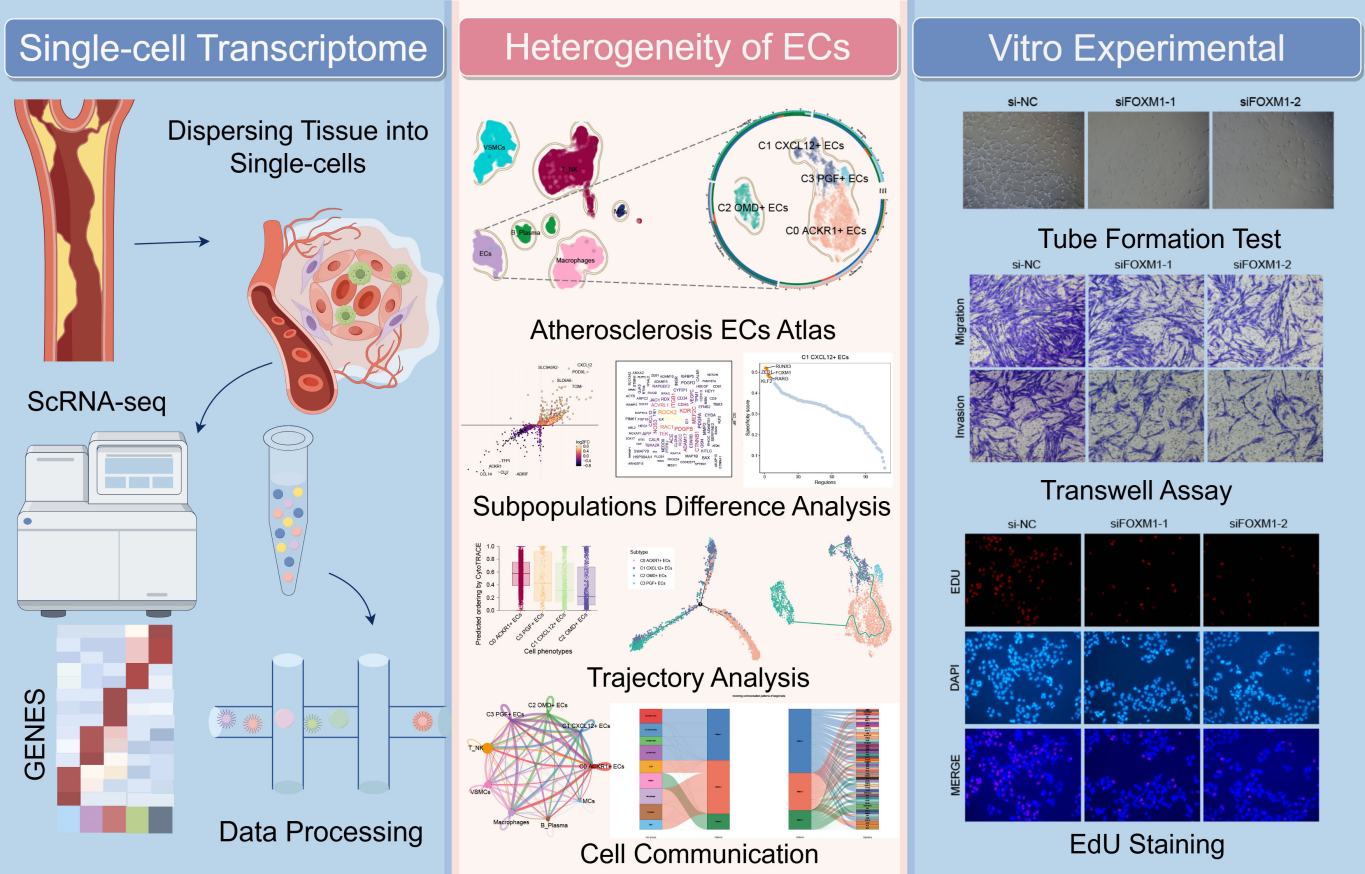
Flow chart of this study. Key Endothelial Cells Characteristics in AS: Single-cell RNA sequencing of 4,823 ECs, combined with analyses using CytoTRACE, Monocle2, Slingshot, and CellChat, identified C1 *CXCL12*+ ECs as a pivotal subpopulation. These cells exhibited high proliferative capacity and were enriched in pathways associated with endothelial migration and repair. This was further validated through *in vitro* experiments.

**Figure 2:**
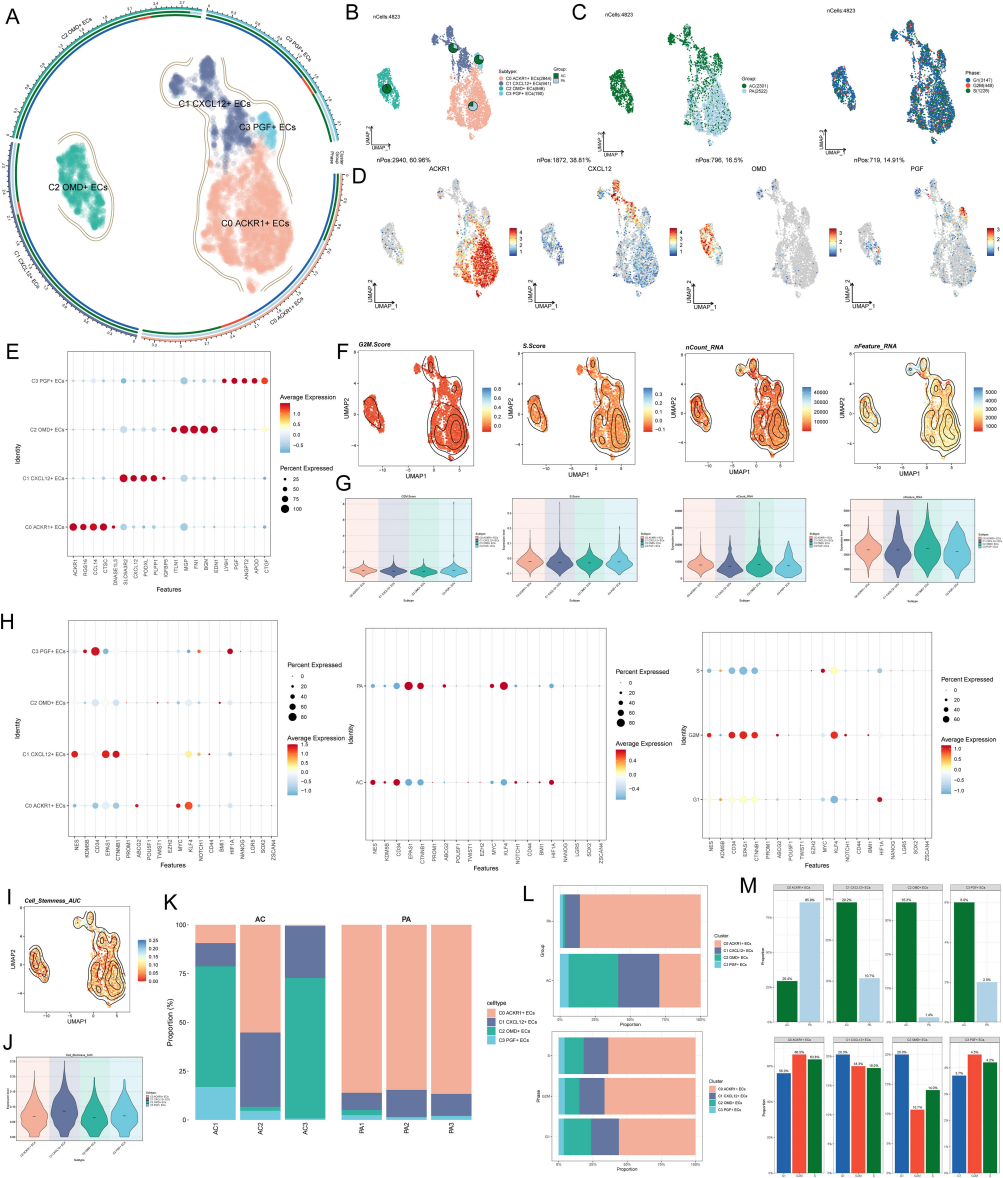
Difference analysis of **Figure 1** AS EC subpopulations. **(A)** The UMAP plot showed that AS ECs were divided into four different cell subpopulations: C0 *ACKR1*+ECS, C1 *CXCL12*+ ECs, C2 *OMD+* ECs and C3 *PGF+* ECs. **(B)** The UMAP plot showed the distribution of four EC subpopulations and the tissue source of each subgroup was visualized by pie chart (AC: the core of AS; PA: the vicinity of AS). **(C)** The UMAP plots described the distribution of different tissue types (AC, PA) and cell cycle stages (G1, G2/M, S) of AS ECs. **(D)** Level of expression of *ACKR1*, *CXCL12*, *OMD* and *PGF* genes in all ECs. **(E)** Bubble diagram showed the expression of TOP5 differential genes in four EC subpopulations. **(F-G)** Contour maps and violin maps showed the difference levels of G2/M.Score, S. Score, nCount RNA and nFeature RNA in AS ECs. **(H)** Bubble diagram showed the differential expression of dry gene set in different EC subpopulations, different tissue types and different cell cycle stages. **(I-J)** Contour map and violin map showed the difference level of Cell Stemness AUC of ECs. **(K)** Stacked bar chart showed the proportion of four EC subpopulations in different samples. **(L-M)** Stacked bar charts and faceted bar charts showed the proportion of four EC subpopulations in different tissue types and different phases.

In order to study the single-cell landscape and molecular characteristics of AS, we used scRNA-seq technology to analyze the single-cell data with sample number GSE159677 (**Figure 1**), and used strict data filtering and dimensionality reduction clustering technology to divide the ECs in AS into four subgroups (**Figure 2A**). According to the cell gene map and typical markers, four EC subgroups were named as C0 *ACKR1*+ECs, C1 *CXCL* 12+ ECs, C2 *OMD*+ ECs and C3 *PGF*+ ECs. The study revealed the histological characteristics of these cells (AC: the core of AS; PA: the vicinity of AS) and cell cycle status (G1, G2/M, S) (**Figures 2B, C**). There were obvious differences in the histological characteristics of these cells. C0 *ACKR1*+ ECs mainly came from PA, C1 *CXCL12*+ ECs and C3 *PGF+* ECs mainly came from AC, and C2 *OMD+* ECs basically all came from AC (**Figure 2B**). However, the value-added characteristics of these cell subpopulations were not obviously biased (**Figure 2C**). The expressions of marker genes *ACKR1, CXCL12, OMD* and *PGF* in four ECs subpopulations were shown in **Figure 2D**. Bubble diagram visualized the TOP5 differential genes of EC subpopulations, and it could be seen that there were obvious differences in gene expression among the four subpopulations (**Figure 2E**). G2/M score, S score, nCount RNA, and nFeature RNA levels were compared among AS EC subpopulations (**Figures 2F, G**). Next, we analyzed the difference of average expression levels among four EC subpopulations, different tissue types and different cell cycle characteristics (**Figure 2H**). The results showed that compared with other subgroups, the expression of the C1 *CXCL12+* ECs dry gene was possibly much more active, indicating that it had greater proliferation ability and was active in the progress of AS disease. Among them, the expression levels of *NES, EPAS1* and *CTNNB1* were higher in C1 *CXCL12+* ECs. In addition, cells derived from PA and in G2/M phase had a high level of stemness and relatively strong ability to increase their value. The Cell Stemness AUC level of C1 *CXCL12*+ EC was high, which was consistent with the previous analysis (**Figures 2I, J**). Finally, the stacked bar charts and faceted bar charts were used to show the proportion of four EC subpopulations in different tissue types and different phases (**Figures 2K, M**). In terms of organizational origin, most of PA were C0 ACKR1+ECSand C1 *CXCL12+* ECs, while most of AC are C0 *ACKR1+*ECs, C1 *CXCL12*+ ECs and C2 *OMD*+ ECs. In addition, there was no significant difference in the distribution of the four ECs subpopulations in each cycle stage.

### Enrichment analysis of ECs

**Figure 3:**
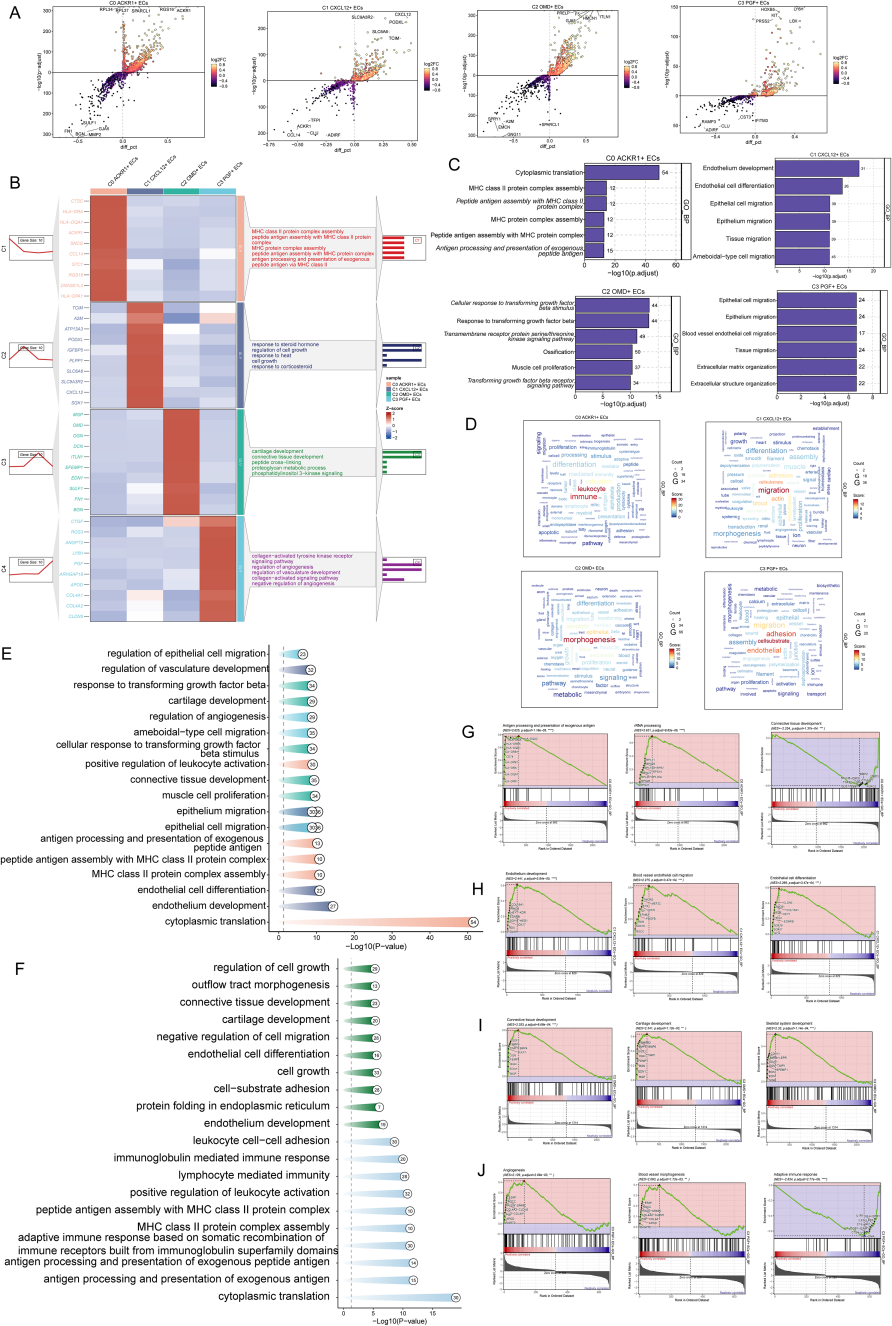
Enrichment analysis of ECs. **(A)** Volcano plots showed up- and down-regulated genes in four EC subpopulations. **(B)** Heatmap demonstrated enrichment analysis of differential genes in EC subpopulations, with the first five pathways displayed in the figure. **(C, D)** Bar graphs and word cloud plots demonstrated the results of GOBP enrichment analysis. **(E, F)** Enrichment analysis in different EC subpopulations as well as different tissue types. **(G, J)** Enrichment analysis of GSEA in four EC subpopulations.

To further understand the biofunctional properties as well as molecular characterization of different EC subpopulations, differential genes of EC subpopulations were analyzed for functional enrichment. Volcano plots demonstrated the up-regulated and down-regulated differential genes in the four EC subpopulations (**Figure 3A**). Further enrichment analysis showed significant differences in enrichment results among different EC subpopulations, with C0 *ACKR1+* ECs enriched for pathways such as Cytoplasmic translation, MHC class II protein complex assembly, Peptide antigen assembly with MHC class II protein complex, MHC protein complex assembly, Peptide antigen assembly with MHC protein complex, Antigen processing and presentation of exogenous peptide antigen; C1 *CXCL12+* ECs were enriched for pathways such as Endothelium development, EC differentiation, Epithelial cell migration, Epithelium migration, Tissue migration, Ameboidal-type cell migration; C2 *OMD+* ECs were enriched for pathways such as Cellular response to transforming growth factor beta stimulus Response to transforming growth factor beta, Transmembrane receptor protein serine/threonine kinase signaling pathway, Ossification, Muscle cell proliferation, Transforming growth factor beta receptor signaling pathway; pathways enriched for C3 *PGF+* ECs were Epithelial cell migration, Epithelium migration, and Blood vessel EC migration, Tissue migration, Extracellular matrix organization, Extracellular structure organization (**Figures 3B, C, E**). The findings from the word cloud analysis aligned with the above observations: C0 *ACKR1+* ECs were enriched in pathways related to leukocyte and immune responses; C1 *CXCL12+* ECs were associated with migration, vessel formation, and actin dynamics; C2 *OMD+* ECs were linked to morphogenesis and epithelial development; and C3 *PGF+* ECs were enriched in cell-substrate adhesion, endothelial function, and migration (**Figure 3D**). These results suggested that C1 *CXCL12*+ ECs and C3 *PGF+* ECs were involved in EC migration and vascular responses during the progression of AS. The enrichment resulted from different tissue types further revealed that cells from the AC region were enriched in pathways such as EC differentiation, endothelium development, and cell growth, while cells from the PA region were associated with immune-related pathways, including leukocyte cell-cell adhesion, immunoglobulin-mediated immune response, lymphocyte-mediated immunity, and positive regulation of leukocyte activation (**Figure 3F**).Subsequent GSEA enrichment analysis showed that C0 *ACKR1*+ ECs were positively enriched in pathways such as antigen processing and presentation of exogenous antigens and rRNA processing, while negatively enriched in connective tissue development (**Figure 3G**). C1 *CXCL12*+ ECs were positively enriched in pathways related to endothelium development, blood vessel EC migration, and EC differentiation (**Figure 3H**). C2 *OMD+* ECs were positively enriched in connective tissue development, cartilage development, and skeletal system development (**Figure 3I**). C3 *PGF+* ECs were positively enriched in angiogenesis, blood vessel morphogenesis, and adaptive immune response (**Figure 3J**). In conclusion, C1 *CXCL12*+ ECs were closely linked to EC differentiation and migration, highlighting them as a highly proliferative subpopulation playing a critical role in the progression of AS.

### TFs analysis

**Figure 4:**
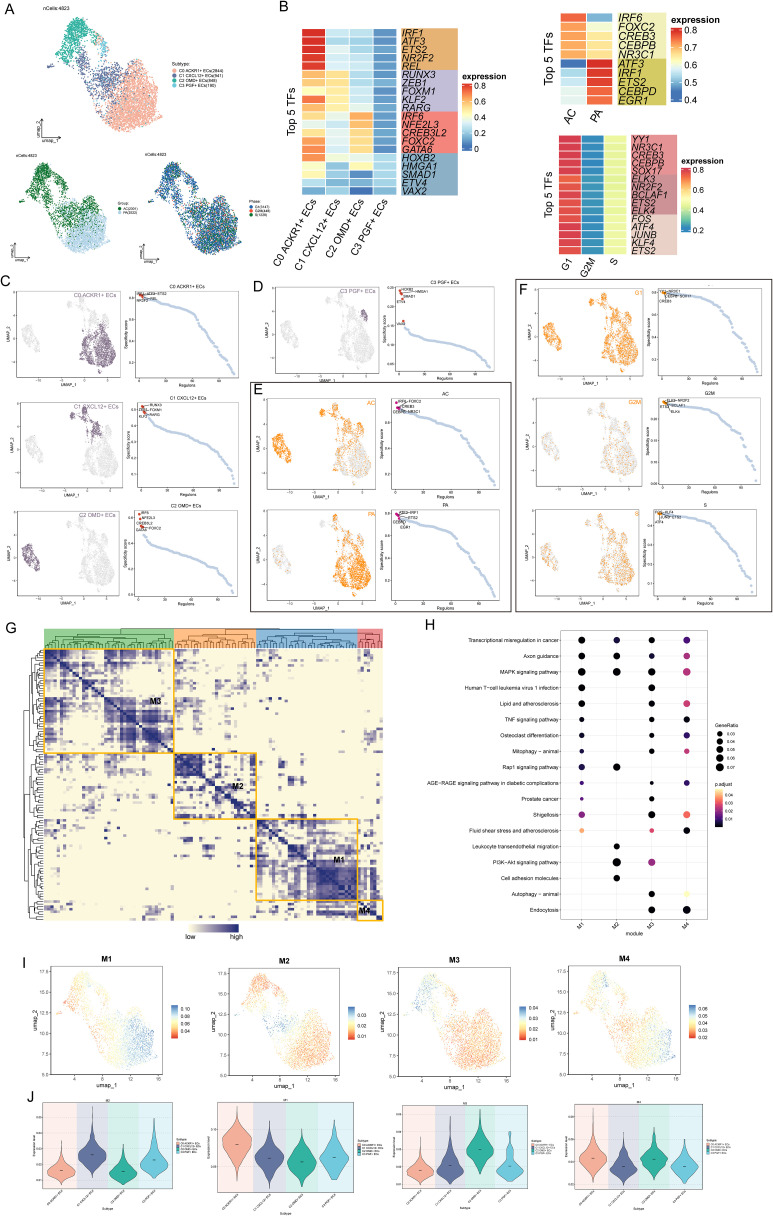
TFs analysis of atherosclerotic EC subsets. **(A)** Based on the expression of TFs in ECs, the cluster analysis was carried out, and the UMAP diagram showed the distribution of cell subgroups, tissue types and time phases. **(B)** The heatmaps showed the expression levels of the top five TFs in four cell clusters, different tissues and different cell cycle phases. **(C, D)** UMAP diagrams showed the distribution of TFs in four EC subpopulations, and the top five TFs in each EC subset were displayed on the right according to the specificity of regulatory factors. **(E, F)** UMAP diagrams showed the distribution differences of TFs in different tissues and different cell cycles, and showed the ranking of the first five TFs in each tissue type and each cell cycle stage on the right. **(G)** Heatmap showed four main TFs modules of ECs -M1, M2, M3 and M4. **(H)** Bubble diagram showed the enrichment of channels in different control modules. **(I, J)** UMAP diagrams and violin diagrams showed the differential expression of different EC subpopulations under the four TF regulation modules.

TFs could directly act on the genome, regulate gene transcription by combining the specific nucleotide sequence upstream of the gene, and affected the biological function of cells. Firstly, we clustered the ECs according to the activity of regulatory factors (**Figure 4A**). UMAP map based on regulatory factor activity had a small degree of dispersion, all ECs were clustered and distributed, C1 *CXCL12*+ ECs was distributed in the middle of EC group, and the tissue properties favored AC. Next, we showed the expression, distribution and rank of TOP5 TFs in four EC clusters, different tissues and different cell cycle phases (**Figures 4B–F**). The TOP5 TFs of C0*ACKR1*+ECS were IRF1, ATF3, ETS2, NR2F2, REL. TOP5 TFs of C1 *CXCL12*+ ECs are RUNX3, ZEB1, FOXM1, KLF2 and RARG. TOP5 TFs of C2 *OMD+* ECs were IRF6, NFE2L3, CREB3L2, FOXC2 and GATA 6, respectively. TOP5 TFs of C3 *PGF+* ECs were HOXB2, HMGA1, SMAD1, ETV4 and VAX2, respectively. It was worth noting that the TFs activity in C0 *ACKR1*+ ECS was generally higher, followed by C1 *CXCL12*+ ECs and C2 *OMD+* ECs. Then, we divided the TFs of ECs into four regulatory modules (M1, M2, M3 and M4) by the connection specificity index (CSL) matrix (**Figure 4G**). The TFs in different regulatory modules were enriched and analyzed. The TFs in M1 and M3 were enriched in transcription al regulation in cancer, MAPK signaling pathway, lipid and AS and TNF signaling pathway. The TFs in M2 were enriched in Leukocyte transendothelial migration, P13K-Akt signaling pathway and cell adhesion molecules. The TFs in M4 were enriched in the fluid shear stress and AS pathways (**Figure 4H**). These modules were mapped to UMAP and violin plots, and the regulatory activities of different EC subpopulations in each module were analyzed (**Figures 4I, J**). In M1 regulatory module, C0 *ACKR1*+ ECS was more active. In M2 regulation module, C1 *CXCL12*+ ECs and C3 *PGF+* ECs were more active; In M3 control module, C2 *OMD+* ECs was active; In M4 control module, C0 *ACKR1*+ ECS and C2 *OMD+* ECS were more active.

### Characterization of TOP5 TFs in C1 CXCL 12+ ECs and metabolic characterization

**Figure 5:**
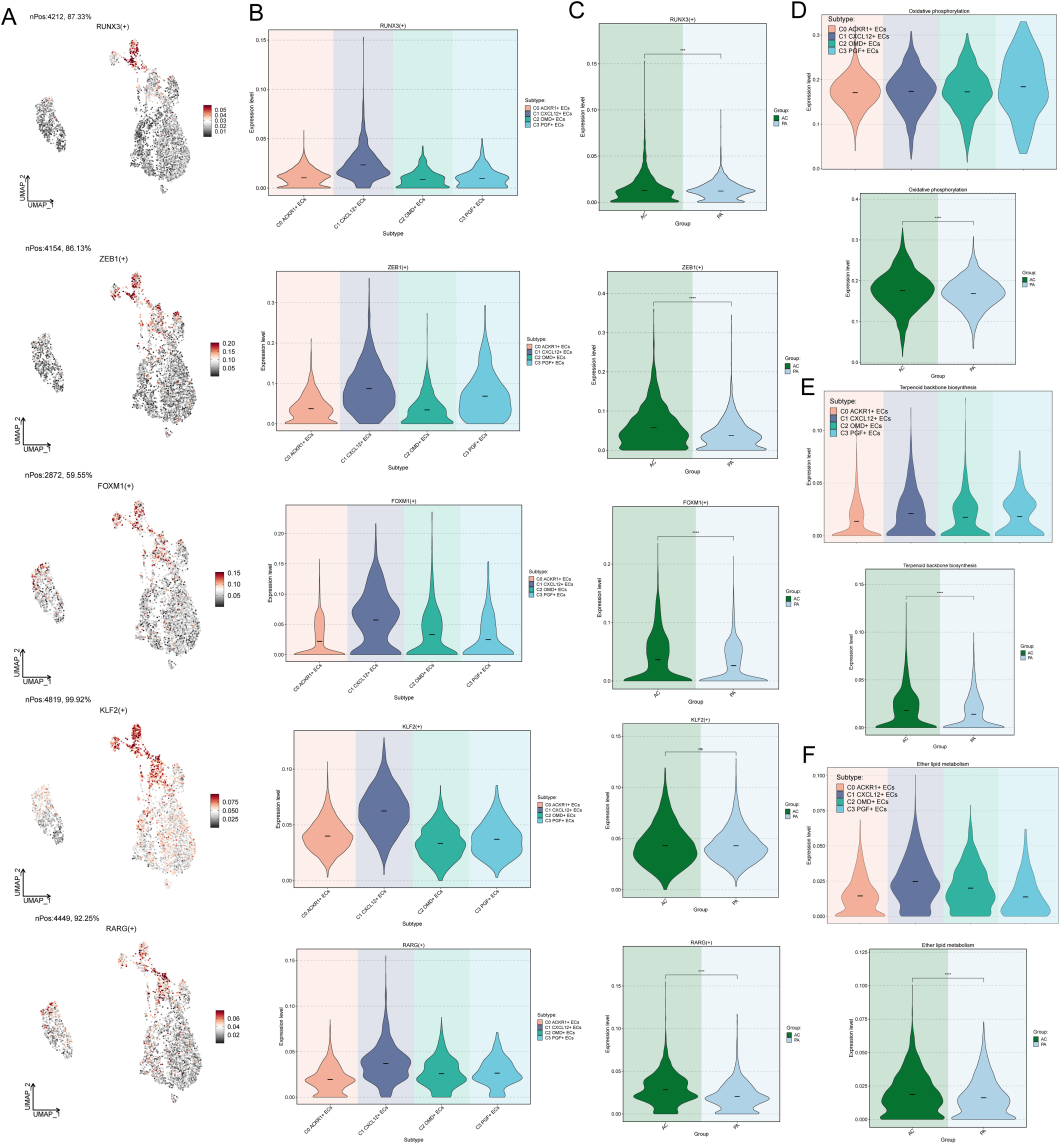
Characterization of TOP5 TFs in C1 CXCL 12+ ECs. **(A)** UMAP plots showed the distribution density of TOP5 TFs RUNX3(+), ZEB1(+), FOXM1(+), KLF2(+), RARG (+) in C1 *CXCL12*+ ECs. **(B)** Violin plots demonstrated differential expression of RUNX3(+), ZEB1(+), FOXM1(+), KLF2(+), RARG (+) in four EC subpopulations. **(C)** Violin plots demonstrated differential expression of RUNX3(+), ZEB1(+), FOXM1(+), KLF2(+), RARG (+) in AC, PA. **(D-F)** violin plots demonstrated the differential regulation of Oxidative phosphorylation, Terpenoid backbone biosynthesis and Ether lipid metabolism metabolic pathways in EC subpopulations as well as in different tissue types. ***p<0.001, **** p<0.0001.

Next, the expression of TOP5 TFs in C1 *CXCL12*+ ECs was analyzed in four EC subpopulations as well as in different tissues (**Figures 5A–C**), in which ZEB1(+), FOXM1(+), and KLF2(+) were more prominently distributed in C1 *CXCL12*+ ECs, and the expression of ZEB1(+), FOXM1(+), RARG (+) were more prominently expressed in ACs.

We further explored cellular metabolism to identify metabolic pathways closely related to ECs. Oxidative phosphorylation, Terpenoid backbone biosynthesis, and Ether lipid metabolism were metabolic pathways associated with ECs, of which Oxidative phosphorylation was active in all EC subpopulations, and similarly all of these metabolic pathways were active in AC-derived cells (**Figures 5D–F**). Taken together, this suggested that Oxidative phosphorylation was a metabolic pathway closely associated with ECs. Oxidative phosphorylation maintained endothelial cell function by generating energy. Excessive reactive oxygen species (ROS) damaged the endothelium, leading to dysfunction and inflammation, and promoting the occurrence of AS.

### Quasitemporal analysis of endothelial cell subsets further revealed the critical role of C1 *CXCL12 +* ECs

**Figure 6:**
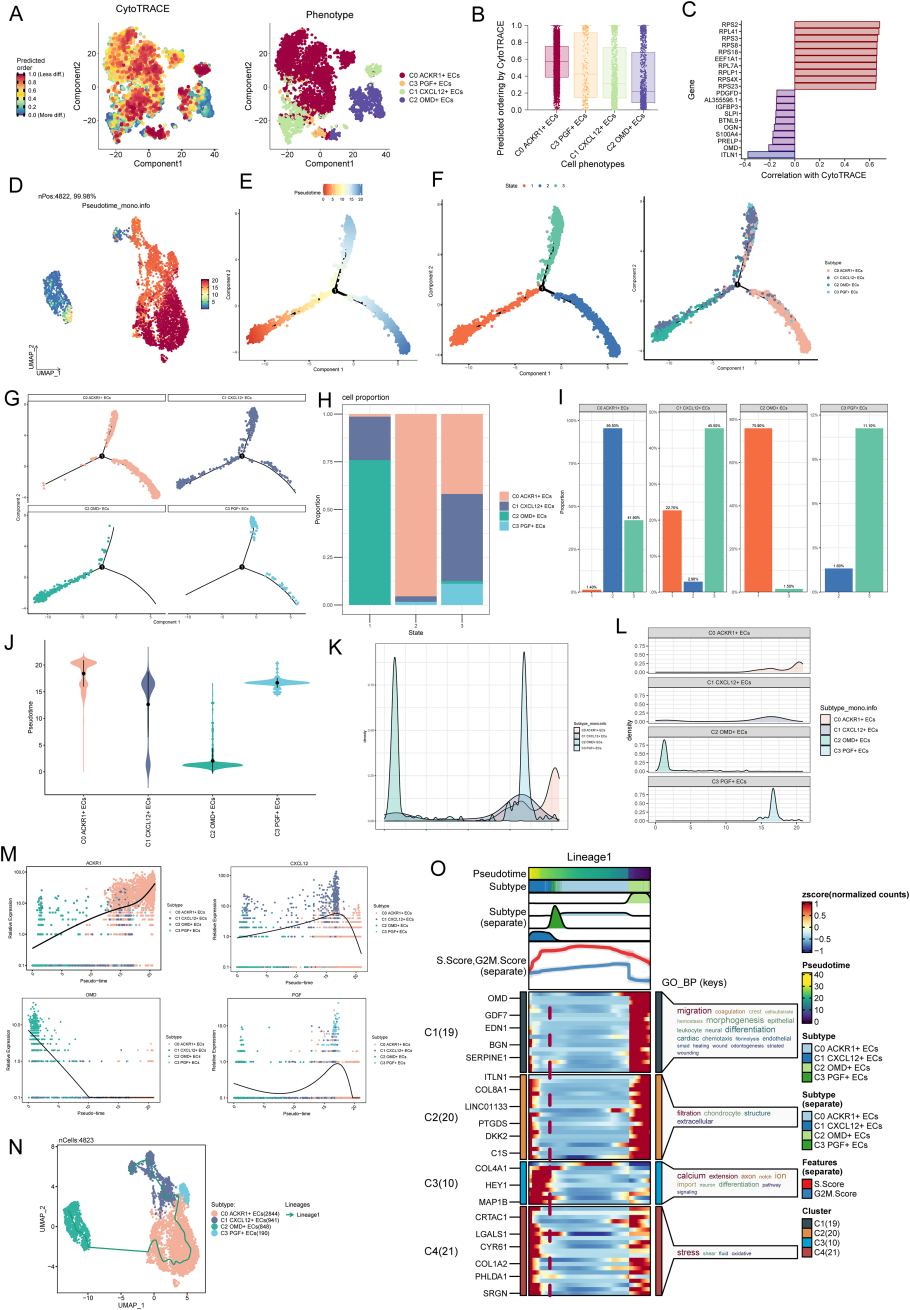
Proposed chronological analysis of EC subpopulations further revealed the critical role of C1 CXCL 12+ ECs. **(A)** Using CytoTRACE, the order of differentiation was inferred for EC subpopulations, and the spatial distribution characteristics of the corresponding cell subpopulations were shown on the right. **(B)** Using CytoTRACE, EC subpopulations were sorted, with higher scores indicating higher stemness levels and greater differentiation potential. **(C)** Bar graph showed the correlation analysis of stemness genes with CytoTRACE. **(D)** UMAP plot showed the differentiation trajectory of putative cell subpopulations using Monocle2. **(E)** Two-dimensional trajectory plot demonstrated the putative cell differentiation trajectory with 1 branch. **(F)** 2D trajectory plots demonstrated the division of the predicted trajectory into 3 States (left) and coloring according to cell subpopulations (right). The temporal trajectories of the predicted subpopulations used the monocle2 reveal 4 distinct trajectories, and the UMAP plot illustrated each trajectory. **(G)** Facet plots showed the distribution of each of the four EC subpopulations on the simulated trajectories. **(H, I)** Bar graphs showed the percentage of different EC subpopulations in the 3 states. **(J)** Violin plots specified the before-and-after order of the four EC subpopulations on the proposed temporal trajectories. **(K, L)** Ridge plots demonstrated the change of the four EC subpopulations with the proposed temporal trajectory. **(M)** Dynamic trend plots demonstrated changes in *ACKR1*, *CXCL12*, OMD and PGF with the proposed temporal trajectory. **(N)** UMAP plot showed a proposed temporal trajectory predicted by simulation using Slingshot. **(O)** GOBP enrichment analysis of Lineage 1.

We used CytoTRACE to analyze the stemness levels of EC subpopulations and to study the value-adding capacity of the cells, C0 *ACKR1*+ ECs had the highest stemness levels and the strongest value-adding capacity, followed by C1 *CXCL12*+ ECs and C3 *PGF+* ECs, which also had strong value-adding capacity, it was worth noting that C2 *OMD+* ECs had the lowest stemness scores (**Figures 6A, B**). In the correlation analysis between stemness genes and CytoTRACE, *OMD* showed a more prominent negative correlation, which might explain the lowest CytoTRACE scores of C2 *OMD+* ECs (**Figure 6C**). In Monocle’s postulated cell differentiation trajectory, there was 1 branching point in the EC differentiation process, which divided the whole process into 3 stages (State 1, State 2, and State 3).The majority of the C2 *OMD+* ECs were distributed in State 1, the majority of the C0 *ACKR1*+ ECs were relatively evenly distributed in State 2 and State 3, C3 *PGF+* ECs were all relatively uniformly distributed in State 2 and State 3, and it was noteworthy that C1 *CXCL12*+ ECs were distributed in all 3 States, but relatively more in State 3 (**Figures 6D–G**). Bar graphs showing the percentage of different EC subpopulations in the 3 States further support the above results, with 75.90% of cells in State 1 originating from C2 *OMD+* ECs, 95.50% of cells in State 2 originating from C0 *ACKR1*+ ECs, and the largest percentage of cells in State 3 being C1 *CXCL12*+ ECs (**Figures 6H, I**). For these four EC subpopulations with the proposed temporal trajectory ordering, C2 *OMD+* ECs were the most forwardly ordered, and it was noteworthy that the proposed temporal ordering positions of C0 *ACKR1*+ ECs, C1 *CXCL12*+ ECs, and C3 *PGF+* ECs were more concentrated and all were in the latter half of the ordering position (**Figures 6J–L**).The marker genes of the four EC subpopulations *ACKR1*, *CXCL12*, *OMD* and *PGF* changed with the proposed temporal trajectory as shown in **Figure 6M**. One differentiation trajectory of ECs was inferred by Slingshot analysis (**Figure 6N**), Lineage1: C2 *OMD+* ECs→C0 *ACKR1*+ ECs→C3 *PGF+* ECs→C1 *CXCL12*+ ECs. Consistent with the previous analysis, C1 *CXCL12*+ ECs were at the terminal stage of the differentiation trajectory. GOBP enrichment analysis of Lineage 1 revealed that C1 *CXCL12*+ ECs were enriched in pathways such as stress and oxidative, and C2 *OMD+* ECs were enriched in pathways such as migration, differentiations, and endothelia (**Figure 6O**). These analyses revealed the stemness levels, differentiation trajectories, and functional enrichment analyses of different endothelial cell subsets. C1 CXCL12+ endothelial cells were enriched in oxidative stress pathways, suggesting that these cells might play an important role in AS, especially in vascular endothelial damage and inflammation.

### Analysis of communication pattern of cell interaction in AS

**Figure 7:**
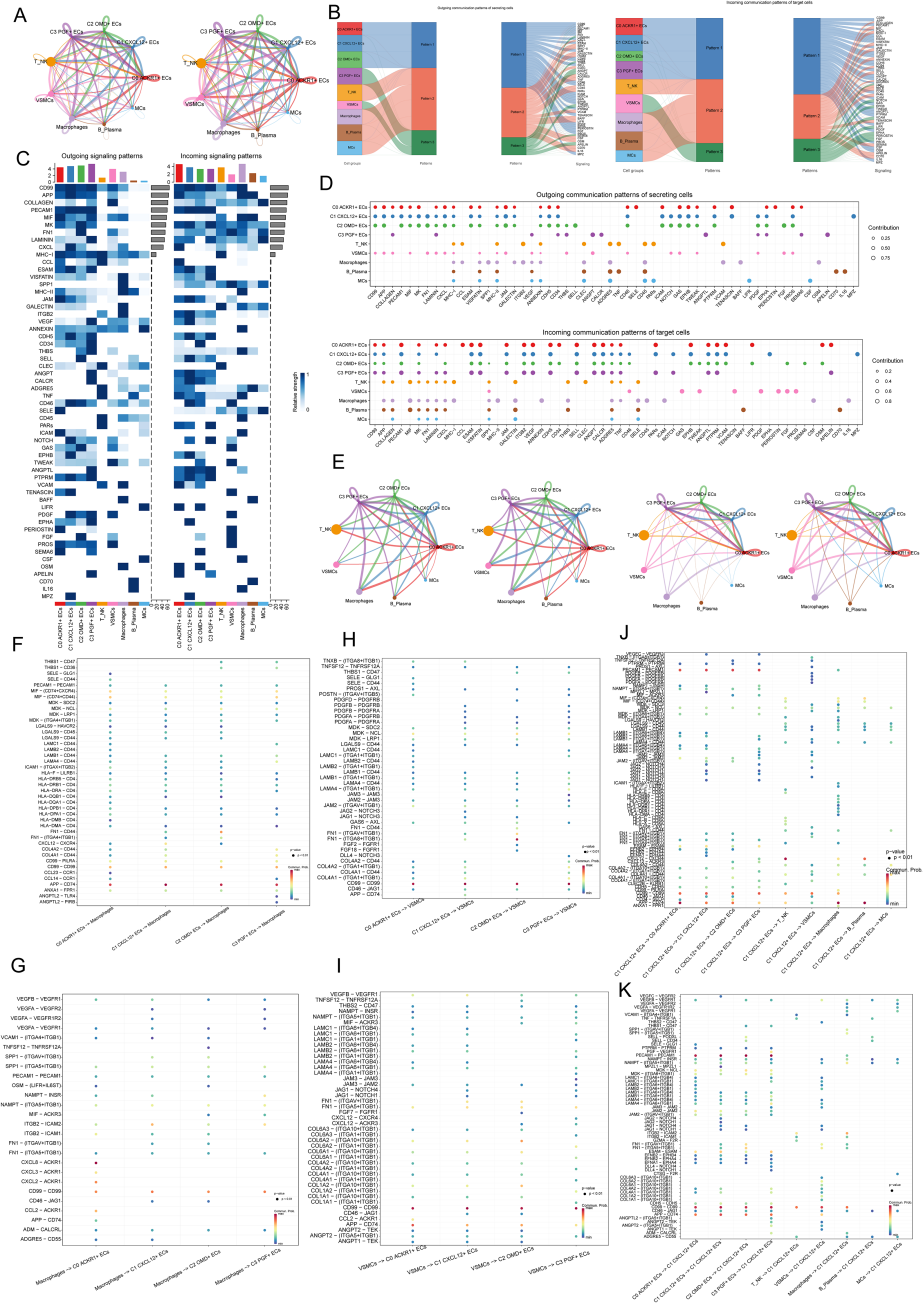
Analysis of communication mode of cell interaction in AS. **(A)** Circles showed the intensity (left) and quantity (right) of the interaction between four EC subpopulations and all other cell clusters in AS. **(B)** Mulberry diagram showed the outward communication patterns of secretory cells (pattern 1, pattern 2 and pattern 3) and the inward communication patterns of target cells (pattern 1, pattern 2 and pattern 3). **(C)** Heatmap showed the strength of various pathways in the output signal mode and the incoming signal mode of all AS cell clusters. The upper bar graph represented the cumulative effect of each cell type in all pathways. **(D)** The bubble diagram showed the role of different types of cells in various pathways in the signal mode. **(E)** The circle diagrams described the intensity and quantitative contribution of the interaction between cells by selecting four EC subpopulations as SOURCE (left) and four EC subpopulations as TARGET (right). **(F, G)** The bubble diagrams showed the receptor ligand pairs that interact between four EC subpopulations and macrophages. **(H, I)** The bubble diagram showed the receptor ligand pairs that interact between four EC subpopulations. **(J, K)** The bubble diagrams showed the interaction between C1 *CXCL12*+ ECs and all other cells.

In order to analyze the cross-talk relationship between AS EC subpopulations and other cells, we showed the number and intensity of interaction between cells by circle diagrams (**Figure 7A**). The results showed that the interaction between four ECs subpopulations and other cells was stronger, and it was also observed that the interaction between EC subpopulations and macrophages and VSMCs was stronger than other cell types, which was reflected in the number and lightness/weight. Then, in order to further analyze the important proteins of incoming signals and outgoing signals between cells, we analyzed the contributions of incoming communication patterns of target cells and outgoing communication patterns of secret cells by using mulberry diagrams, heatmaps and bubble diagrams (**Figures 7B–D**). **Figure 7B** revealed three outward communication patterns of secretory cells (pattern 1, pattern 2 and pattern 3) and three inward communication patterns of target cells (pattern 1, pattern 2 and pattern 3) respectively. Specifically, in the efferent communication pattern of secretory cells, C0 *ACKR1*+ ECs, C1 *CXCL12*+ ECs, C2 *OMD+* ECs interacted with other cells through pattern 1, and the main pathways were CD99, APP, MK, CXCL, etc. T&NK, Macrophages, B&Plasma and MCs interacted with other cells through pattern 2, and the main pathways were CCL, SPP1, etc. C3 *PGF+* ECs and VSMCs interacted with others through pattern 3, and the main pathways were APELIN, LAMININ, etc. In the afferent communication pattern of target cells, all EC subpopulations played a role through pattern 1, and the main proteins were CD99, APP, PECAM1, etc. T&NK, Macrophages, B&Plasma and MCs played a role through pattern 2, and the main proteins were COLLAGEN, MIF, MHC-I, etc. VSMCs played a role through FGF and EPHA in Pattern 3. Because we found that C1 *CXCL12*+ ECs was the key EC subgroup in the progression of AS disease in the previous analysis, we focused on the interaction between C1 *CXCL12*+ ECs and other cells in the subsequent analysis, in order to summarize the important functional proteins. In **Figures 7B, C**, we found that CD99, APP, PECAM1 and CXCL signal pathways contributed greatly to C1 *CXCL12*+ ECs mainly through interaction with other cells. Next, the circle diagram was used to describe the intensity and quantity contribution of the interaction between cells by selecting four ECs subpopulations as SOURCE, and the intensity and quantity contribution of the interaction between cells by selecting four EC subpopulations as TARGET (**Figure 7E**). The receptor ligand paired that contribute greatly to the interaction between EC subpopulations and macrophages were APP-CD74, CXCL8-*ACKR1* and CD99-CD99(**Figures 7F, G**). The receptor ligand paired that contributed greatly to the interaction between EC subpopulations and vascular smooth muscle cells were CD99-CD99, FN1-(ITGA8+ITGB1) and APP-CD74 (**Figures 7H, I**). Finally, we studied the interaction between C1 *CXCL12*+ ECs and all other cells, and found thatApp-CD74 and CD99-CD99 contributed a lot (**Figures 7J, K**). To sum up, APP, CD99 and CXCL important pathways for ECs to interact with other cells, which were related to AS.

### C1 *CXCL12+* ECs plays a strong role in APP signal path and CD99 signal path

**Figure 8:**
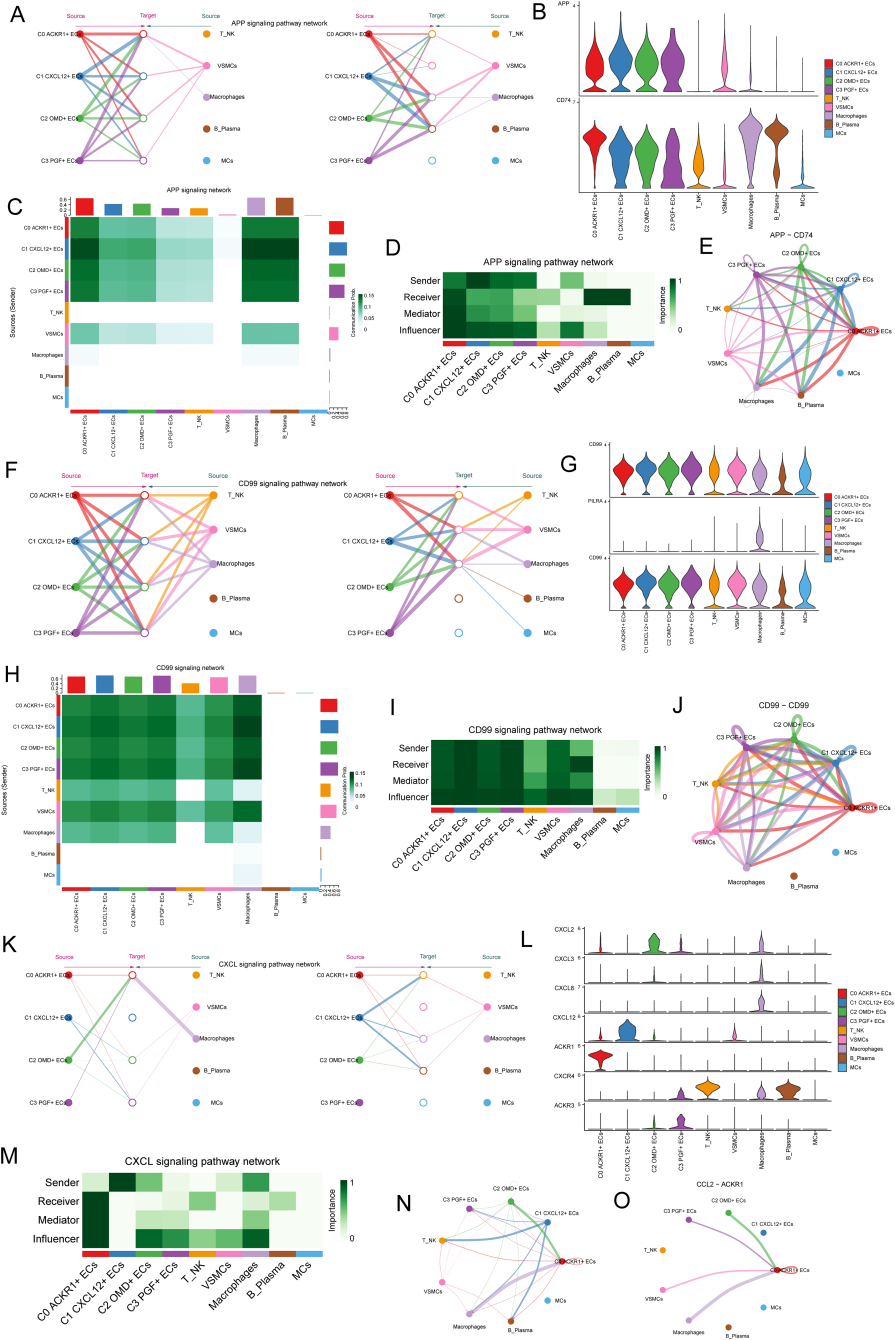
C1 *CXCL12* +ECs plays a strong role in APP signal path, CD99 signal path and CXCL signal path. **(A)** The hierarchical plot showed the autocrine and paracrine interactions of four EC subpopulations, T NK cells, VSMCs, macrophages, B Plasma and MCs in APP signaling pathway. **(B)** Violin diagram showed the proteins interacting between cells in APP signaling pathway. **(C)** Heatmap showed the communication probability of different cell clusters based on APP signaling pathway. **(D)** Heatmap showed the network centrality score of the interaction of various cell types under APP signaling pathway. **(E)** Circle diagram showed the interaction of APP-CD74 protein pairs under APP signaling pathway. **(F)** The hierarchical plot showed the autocrine and paracrine interactions between four EC clusters and other cells in CD99 signaling pathway. **(G)** Violin diagram showed the proteins interacting between cells in CD99 signaling pathway. **(H)** Heatmap showed the communication probability of different cell clusters based on CD99 signaling pathway. **(I)** Heatmap showed the network centrality score of the interaction of various cell types under CD99 signaling pathway. **(J)** Circle diagram showed the interaction of CD99-CD99 protein pair. **(K)** Hierarchical diagram showed the interaction of 4 EC subpopulations with other cells in the CXCL signaling pathway. **(L)** Violin diagram showed proteins interacting between cells in the CXCL signaling pathway. **(M)** Centrality network regulation under the CXCL signaling pathway. **(N-O)** Circle diagram demonstrated the CXCL signaling pathway and the interactions between cells in the CCL2-ACKR1 protein pair.

After revealing the signal pathways of cell interaction, next, we willed study the signal pathways between C1 *CXCL12*+ ECs, macrophages and VSMCs. First of all, we studied the APP signaling network, and the results showed that C1 *CXCL12*+ ECs and VSMCs interacted strongly in the APP signaling pathway, among which the protein pair was APP-CD74 (**Figures 8A, B**). The communication probability of different cell clusters based on APP signaling pathway was shown in **Figure 8C**. In the centrality score, C1 *CXCL12*+ ECs mainly played the role of Sender, macrophage mainly played the role of Receiver, and VSMCs mainly played the role of influencer (**Figure 8D**). The circle diagram showed the interaction of APP-CD74 protein paired under APP signaling pathway (**Figure 8E**), and the results were consistent with the above.

Then, the CD99 signaling network was studied. The results showed that C1 *CXCL12*+ ECs interacted strongly with VSMCs and macrophages in the CD99 signaling pathway, among which CD99, PILRA and CD99 acted mainly through the CD99-CD99 protein pair (**Figures 8F, G**). The communication probability of different cell clusters based on CD99 signaling pathway was shown in **Figure 8H**, and the communication probability of macrophages was higher. In the centrality score, C1 *CXCL12*+ ECs mainly played the roles of Sender, Receiver, Mediator and Influencer, macrophages mainly played the roles of Receiver and Influencer, and VSMCs mainly played the role of influencer (**Figure 8I**). The circle diagram showed the interaction of CD99-CD99 protein pair under CD99 signaling pathway (**Figure 8J**).

Finally, based on CXCL signaling network, the results showed that C1 *CXCL12*+ ECs plays a role (**Figures 8K, L**) through *ACKR1*. The network centrality score showed that C1 *CXCL12*+ ECs mainly played the role of Sender, and macrophages mainly played the role of Influencer (**Figure 8M**). Circle diagram showed the crosstalk between cells in CXCL signal pathway and the interaction of CCL2-ACKR1 protein paired in CXCL signal pathway (**Figures 8N, O**).

### Experimental verification of FOXM1 *in vitro*

**Figure 9:**
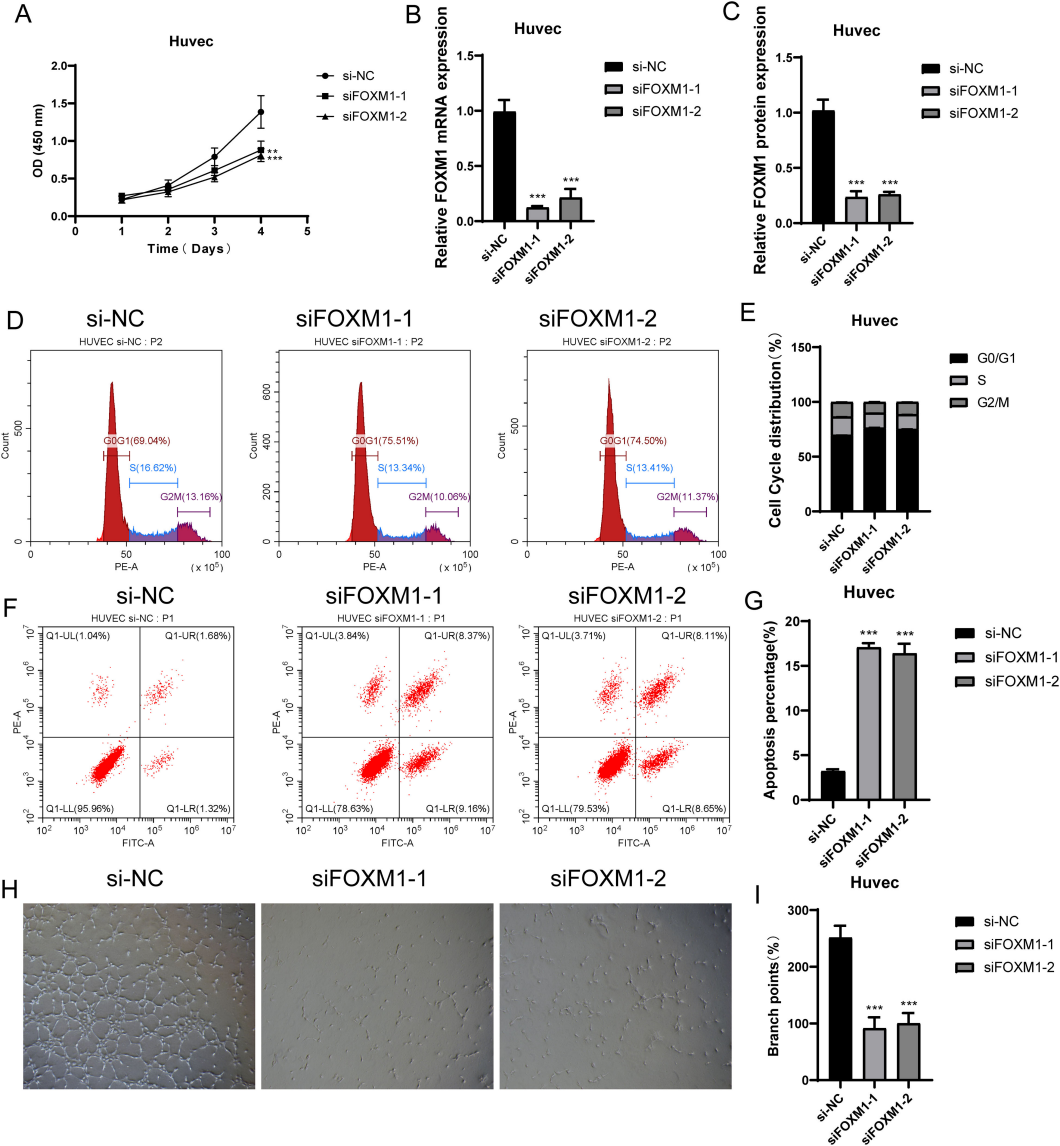
Experimental verification of FOXM1 *in vitro*. **(A)** CCK-8 test showed the comparison of cell viability before and after FOXM1 knockout. **(B, C)** qRT-PCR was used to detect the mRNA and protein expression levels in ECs before and after FOXM1 knockout. **(D, E)** Changes of cell cycle stages (G0/G1, S, G2/M) before and after FOXM1 knockout. **(F, G)** Effect of FOXM1 knockout on apoptosis rate. **(H, I)**. The results of tube-forming experiment verified the functional changes of ECs before and after FOXM1 knockout. **p<0.01, ***p<0.001.

**Figure 10:**
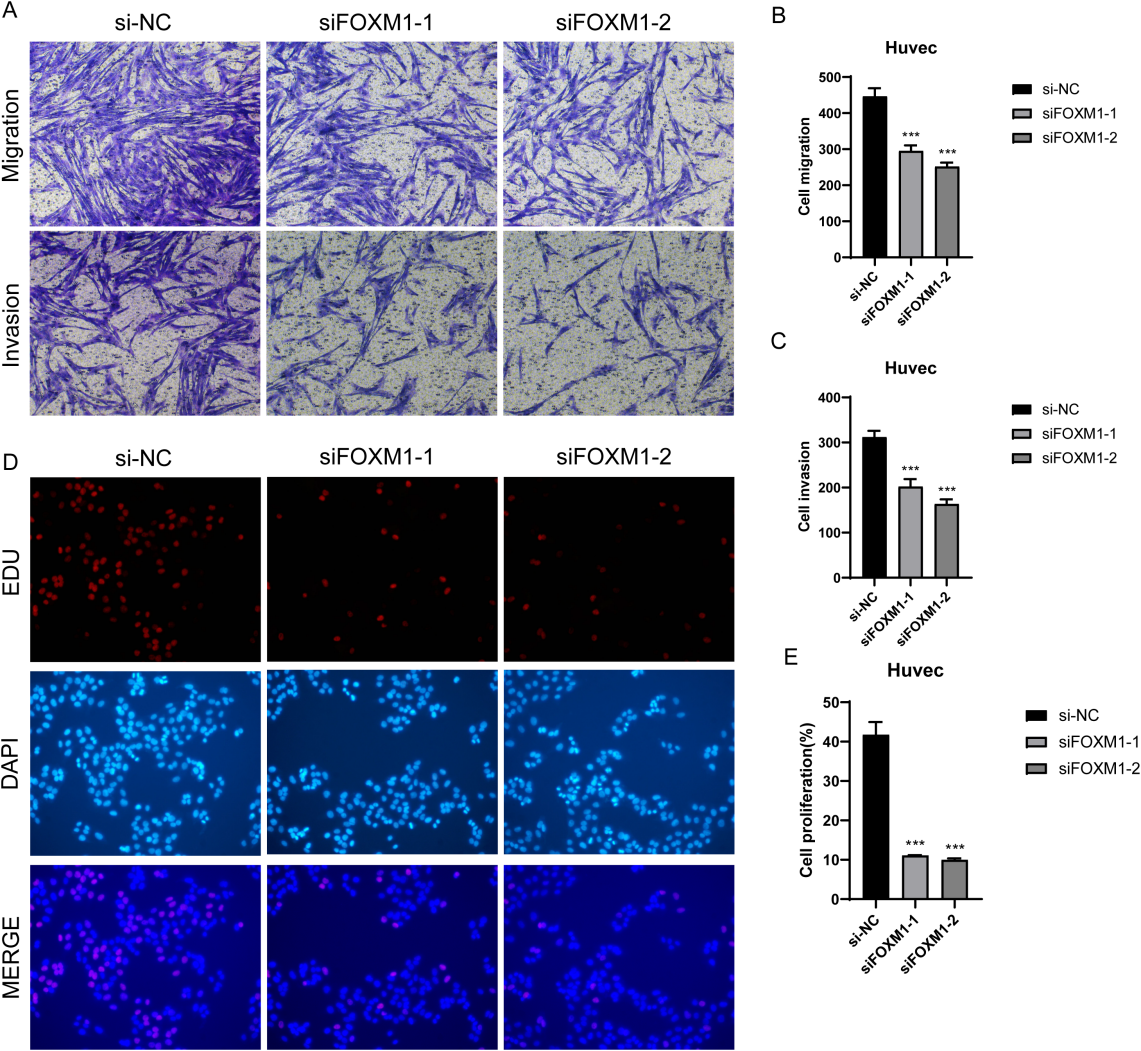
Effect of FOXMI on proliferation, invasion and metastasis of ECs. **(A-C)** Transwell experiment showed that the migration and invasion of Huvec cells were significantly reduced after FOXM1 knockout. **(D-E)** The results of EdU staining showed that the proliferation of Huvec cells was inhibited after FOXM1 knockout. ***p<0.001.

In the previous analysis, we found that C1 *CXCL12*+ ECs had high value-added ability, and it was at the back of pseudo-sequential stage, which was related to EC migration and vascular response, and also played an important role in oxidative stress. FOXM1 was the TOP3 TF of C1 *CXCL12*+ ECs. We studied the effect of C1 *CXCL12*+ ECs on the migration and proliferation of ECs in AS by knocking out FOXM1 gene. We chose Huvec cell line for the experiment, and used the methods of comparing negative control and knocking out FOXM1 group. In the cell viability test (**Figure 9A**), CCK-8 test showed that the cell viability was significantly reduced after knockout. QRT-PCR detection showed that the expression levels of mRNA and protein in ECs were significantly decreased after FOXM1 knockout (**Figures 9B, C**). Compared with the negative control group, the proportion of cells in G0/G1 cell cycle after FOXM1 knockout was relatively increased (**Figures 9D, E**). In addition, the apoptosis rate increased significantly after FOXM1 knockout (**Figures 9F, G**). We further verified the functional changes of ECs after FOXM1 knockout by the results of tube-forming experiment, and the angiogenesis ability of FOXM1 knockout group decreased significantly (**Figures 9H, I**). Finally, the results of Transwell experiment (**Figures 10A–C**) and EdU staining (**Figures 10D, E**) showed that the proliferation, migration and invasion of Huvec cells were significantly reduced after FOXM1 knockout. Therefore, through these experiments, we found that the knockdown of FOXM1 in ECs led to the decrease of proliferation, migration and invasion, thus impacting the progress of AS.

## Discussion

AS was a chronic inflammatory disease characterized by the accumulation of lipids and immune cells within the arterial walls, leading to plaque formation and vascular dysfunction. Despite extensive research, the cellular and molecular mechanisms underlying AS progression remain incompletely understood. Our study leveraged scRNA-seq to dissect the heterogeneity of ECs in AS. By integrating multiple bioinformatics approaches, we identified key EC subpopulations, their transcriptional profiles, regulatory pathways, and potential roles in AS pathogenesis (67).

Using scRNA-seq (68) data from the Gene Expression Omnibus (GEO) database (GSE159677), we applied strict quality control and dimensionality reduction methods to identify four distinct EC subpopulations: C0 *ACKR1*+ ECs, C1 *CXCL12*+ ECs, C2 *OMD+* ECs, and C3 *PGF+* ECs.

This study investigated cellular heterogeneity in relation to disease progression, proliferative stemness, metabolic activity, and interactions with other cells. By employing single-cell analysis methods such as CytoTRACE, Monocle2, and Slingshot, we inferred the differentiation trajectories and molecular characteristics of EC subpopulations along pseudo-time. These analyses identified a specific EC subpopulation, C1 *CXCL12*+ ECs, as the focus of our study. The selection of this subpopulation was based on several critical factors.

Firstly, the distribution of these subpopulations across tissue regions (AC and PA) highlighted the tissue-specific heterogeneity of ECs. Remarkably, C0 *ACKR1*+ ECs predominantly originated from the PA region, while C1 *CXCL12*+ ECs and C3 *PGF+* ECs were enriched in the AC region. The distinct expression patterns of the marker genes *ACKR1*, *CXCL12*, OMD, and PGF underscored their critical roles in defining subpopulation-specific functions.

Secondly, DEGs and pathway enrichment analyses revealed unique biological roles for each endothelial subpopulation (69). instance, C0 *ACKR1*+ ECs were enriched in antigen processing and immune response pathways, while C1 *CXCL12*+ ECs showed strong associations with endothelial development, migration, and differentiation pathways. C2 *OMD+* ECs were linked to transforming growth factor-β (TGF-β) signaling and ossification, whereas C3 *PGF+* ECs were involved in extracellular matrix organization and vascular morphogenesis. These findings suggested that C1 *CXCL12*+ ECs and C3 *PGF+* ECs might play pivotal roles in endothelial migration and vascular remodeling during AS progression.

Furthermore, using the AUCell and CytoTRACE methods, we assessed the stemness and proliferative potential of the endothelial subpopulations. C1 *CXCL12*+ ECs displayed higher stemness and proliferative activity compared to other subpopulations, supported by elevated expression of stemness-associated ECs. Cells from PA regions and those in the G2/M cell cycle phase also exhibited increased stemness levels, indicating a dynamic interplay between tissue localization, EPAS1, and CTNNB1. dynamic interplay between tissue localization, cell cycle stage, and EC function in AS.

More importantly, in the putative temporal trajectory analysis, C1 *CXCL12*+ ECs were revealed to be at the end of the trajectory, in a subpopulation important for late AS progression. Specifically, in the EC trajectories analyzed by Monocle2, the largest percentage of cells in State 3 were C1 *CXCL12*+ ECs. consistently, in the Slingshot analysis, C1 *CXCL12*+ ECs were at the terminal stage of the Lineage1 differentiation trajectory. Enrichment analysis revealed that C1 *CXCL12*+ ECs were enriched in pathways such as stress and oxidative. Previous studies had found that one of the major stimulators of angiogenesis was hypoxia, which was frequently observed in disease settings such as cancer and AS (70). Hypoxia induces HIF-1α to bind to the promoter region of CXCL12, enhance its transcription level, and activate the CXCL12/CXCR4 axis, thereby promoting the repair function of endothelial cells and angiogenesis, which plays a key role in the remodeling process of AS lesions. So, it was further confirmed that C1 *CXCL12*+ ECs might be associated with hypoxia as a key cellular subset affecting AS.

According to previous studies, *CXCL12* was a key regulator in the progression of AS (71). CXC chemokine ligand 12 (*CXCL12*) was a chemokine that played a critical role in cell chemotaxis, proliferation, migration, and metabolism (72). *CXCL12*-CXCR4 axis played a complex role in the pathologic progression of AS and vascular repair, affecting both monocyte migration and adhesion and maintaining vascular homeostasis by regulating the interaction of EPCs and SMCs (73). targeting *CXCL12* might help to reduce inflammation, lipid dysregulation and plaque formation while restoring vascular homeostasis (74).

SCENIC analysis identified key TFs governing the activity of the endothelial subpopulations. For C1 *CXCL12*+ ECs, the top five TFs with the highest activity were RUNX3, ZEB1, FOXM1, KLF2, and RARG. RUNX3 and ZEB1 were known to regulate endothelial differentiation and migration (75), while FOXM1 was essential for cell proliferation and vascular repair. KLF2, a critical regulator of endothelial homeostasis, and RARG, involved in retinoic acid signaling, further emphasize the unique regulatory framework supporting the activity of C1 *CXCL12*+ ECs. The differential activity of these TFs aligned with the proliferative and migratory properties observed in this subpopulation, suggesting their involvement in driving vascular remodeling and inflammation in AS. Future experiments, such as chromatin immunoprecipitation sequencing (ChIP-seq), were needed to validate the direct targets of these TFs and their functional roles.

ZEB1, a key TF of the ZEB family, regulates angiogenesis, endothelial dysfunction, monocyte-EC interactions (76), and contributes to atherosclerotic plaque stability by affecting lipid accumulation (77), macrophage polarization, and VSMC functions (78). ZEB1 also indirectly regulated the immune-inflammatory response, further preventing the progression of AS (79). ZEB1 was an important angiogenic regulator that promotes vascular EC proliferation (80). PSMB8-AS1 promoted vascular inflammation and AS via the NONO/PSMB9/ZEB1 axis (81).

RUNX3 was an important regulator of angiogenesis and cell proliferation, which restricted EC proliferation, migration and tube-forming ability by inhibiting the expression of VEGF and MMP-9 (82, 83). In addition, RUNX3 was targeted and inhibited by miR-210 under hypoxic conditions, which significantly promoted angiogenesis and invasiveness (84). FOXM1 was a TF that plays a key role in the regulation of cell cycle and proliferation and played a complex role in AS (85). FOXM1 mediated inflammatory responses and increased the secretion of pro-inflammatory cytokines, which further exacerbated the inflammatory milieu of AS (86). KLF2 suppressed ECs through the regulation of KLF2 was sensitive to laminar shear and its increased expression inhibits EC dysfunction, thereby protecting the vasculature (87). RARG inhibited abnormal EC proliferation through the regulation of cell cycle genes and inflammatory signaling, maintaining vascular endothelial integrity (88).

Metabolic analysis and CellChat-based intercellular communication studies revealed critical insights into the metabolic heterogeneity and signaling interactions among endothelial subpopulations. Differential metabolic profiles likely supported distinct functional states, while ligand-receptor interactions identified through CellChat highlight the contribution of intercellular signaling to AS pathways. Receptor for ligand interactions identified through CellChat highlight the contribution of intercellular signaling to AS pathophysiology.

Interacting proteins that played important roles in AS EC subpopulations were APP, CD99, and CXCL. APP was a transmembrane protein that accumulates in atherosclerotic plaques and exacerbates EC dysfunction and inflammatory responses (89). APP enhanced atherosclerotic plaque instability by promoting macrophage inflammatory responses and foam cell formation (90). CD99 was a transmembrane molecule that regulated the process of leukocyte transendothelial migration (TEM). Its expression on ECs and leukocytes promoted the recruitment of monocytes to the endothelium, exacerbating the inflammatory response (91).Over-expression of CD99 disrupted the EC junctions and exacerbates vascular permeability, thereby accelerating the formation of AS (92).

CXCL family proteins (e.g., *CXCL12*, CXCL1) regulated endothelial, smooth muscle and immune cell functions by binding to their receptors (e.g., CXCR4, CXCR2) (93).*CXCL12* recruited endothelial progenitor cells (EPCs) to the site of injury and promotes vascular repair (94). *CXCL12* mediated the migration of monocytes and T-cells via CXCR4, exacerbating the inflammatory response (95).Activation of *CXCL12*/CXCR4 signaling induced aberrant proliferation of VSMCs, which promoted the thickening of atherosclerotic plaques (96).Therefore, our study once again summarized the role of APP, CD99, and CXCL proteins in AS disease. influence multiple cellular interactions in AS disease.

Our findings underscored the complexity of EC heterogeneity in AS and the central role of specific subpopulations in disease progression. The identification of C1 *CXCL12*+ ECs as a highly active and proliferative subpopulation suggested their potential as therapeutic targets for modulating. By identifying key EC subsets and their regulatory networks, this study reveals potential targets for pharmacological intervention and provides a theoretical basis for individualized treatment strategies for AS. In the future, the results of this research are expected to be used to develop precision treatment methods targeting the CXCL12 pathway and promote the application of precision medicine in cardiovascular diseases. Future studies integrating spatial transcriptomics and functional assays were warranted to validate these findings and explore their translational potential.

## Conclusion

This study provided a comprehensive single-cell landscape of ECs in AS, revealing critical subpopulations, pathways, and regulatory factors involved in disease progression. C1 CXCL12+ ECs were a key subset associated with endothelial differentiation, vascular remodeling, and inflammation. These cells exhibited high proliferative potential and are enriched in pathways related to endothelial migration and repair. In addition, our study again summarized the role of APP, CD99 and CXCL proteins in AS disease. These insights contributed to a deeper understanding of AS biology and might guide the development of targeted therapies for this prevalent disease. therapies for this prevalent vascular disease.

## Data Availability

The original contributions presented in the study are included in the article/supplementary material. Further inquiries can be directed to the corresponding author.
